# T1 reactivity as an imaging biomarker in myocardial tissue characterization discriminating normal, ischemic and infarcted myocardium

**DOI:** 10.1007/s10554-019-01554-4

**Published:** 2019-05-15

**Authors:** Marly van Assen, Randy van Dijk, Dirkjan Kuijpers, Rozemarijn Vliegenthart, Matthijs Oudkerk

**Affiliations:** 1 Center for Medical Imaging, University Medical Center Groningen, University of Groningen, Hanzeplein 1, EB 45 Groningen, The Netherlands; 2 Department of Cardiovascular Imaging, HMC-Bronovo, The Hague, The Netherlands; 3 Faculty of Medical Sciences, University of Groningen, Groningen, The Netherlands; 4 Institute for Diagnostic Accuracy, Groningen, The Netherlands

**Keywords:** Cardiac MR—cardiac magnetic resonance imaging, Native T1-mapping, MOLLI—modified look-locker inversion, Adenosine, CAD—coronary artery disease

## Abstract

To demonstrate the potential for differentiating normal and diseased myocardium without Gadolinium using rest and stress T1-mapping. Patients undergoing 1.5T magnetic resonance imaging (MRI) as part of clinical work-up due to suspicion of coronary artery disease (CAD) were included. Adenosine stress perfusion MRI and late gadolinium enhancement (LGE) imaging were performed to identify ischemic and infarcted myocardium. Patients were retrospectively categorized into an ischemic, infarct and control group based on conventional acquisitions. Patient with both ischemic and infarcted myocardium were excluded. A total of 64 patients were included: ten with myocardial ischemia, 15 with myocardial infarction, and 39 controls. A native Modified Look-Locker Inversion Recovery (MOLLI) T1-mapping acquisition was performed at rest and stress. Pixel-wise myocardial T1-maps were acquired in short-axis view with inline motion-correction. Short-axis T1-maps were manually contoured using conservative septal sampling. Regions of interest were sampled in ischemic and infarcted areas detected on perfusion and LGE images. T1 reactivity was calculated as the percentage difference in T1 values between rest and stress. Remote myocardium was defined as myocardium without defects in the ischemic and infarcted group whereas normal myocardium is found in the control group only. Native T1-values were significantly higher in infarcted myocardium in rest and stress [median 1044 ms (interquartile range (IQR) 985–1076) and 1053 ms (IQR 989–1088)] compared to ischemic myocardium [median 961 ms (IQR 939–988) and 958 ms (IQR 945–988)]. T1-reactivity was significantly lower in ischemic and infarcted myocardium [median 0.00% (IQR − 0.18 to 0.16) and 0.41% (IQR 0.09–0.86)] compared to remote myocardium [median 3.54% (IQR 1.48–5.78) and 3.21% (IQR 1.95–4.79)]. Rest-stress T1-mapping is able to distinguish between normal, ischemic, infarcted and remote myocardium using native T1-values and T1-reactivity, and holds potential as an imaging biomarker for tissue characterization in MRI.

## Introduction

Cardiac magnetic resonance imaging (cardiac MR) is currently the reference imaging technique for the evaluation of cardiac function and myocardial viability. In clinical practice, the visual assessment of first pass perfusion of gadolinium-based contrast agents is used to assess the presence of perfusion defects. However, cardiac MR also provides the unique possibility to assess tissue relaxation times, which can potentially be used for tissue characterization. Native T1-mapping can potentially be used to detect myocardial perfusion defects by quantifying myocardial water content without the use of gadolinium as a contrast agent. The water content is correlated to the myocardial blood volume (MBV) [1]. Previous studies have shown that the MBV is altered in diseased myocardium [2]. Due to these alterations, T1-mapping could potentially be used for myocardial tissue characterization during myocardial perfusion cardiac MR by discriminating between normal, ischemic and infarcted myocardium.

However, T1-mapping remains a challenge due to the high variability of reference values that result from differences in magnetic field strength, manufacturer, the type of mapping sequence, and inter-patient differences such as age and gender [3, 4]. One way to reduce this variability is using relative T1 values in calculating T1-reactivity, instead of absolute T1 values. T1-reactivity is defined as the difference between native rest and native stress T1 values, and can be expressed either as an absolute difference (∆T1) or as a percentage of change (T1-reactivity) [5, 6].

A proof of concept study by Liu et al. showed that T1-reactivity was able to differentiate between normal, ischemic, infarcted and remote myocardium in a small group of healthy controls and patients with known coronary artery disease (CAD) [6]. However, this study included patients with a combination of ischemic and infarcted myocardium; consequently, there is a lack of data on the T1-mapping values that result from isolated ischemic and infarcted myocardium. In this study, we further explore the possibilities of native rest-stress T1-mapping as a gadolinium-free method for tissue characterization by discriminating normal, infarcted, ischemic, and remote myocardium among a patient group at intermediate-high risk for CAD.

## Materials and methods

### Patients

All subjects gave written informed consent to participate in the study and the local ethical committee granted approval for the study procedures. Symptomatic patients with suspicion of CAD underwent adenosine myocardial perfusion MR as part of their clinical work up. All patients avoided potential adenosine agonists for at least 24 h prior to the cardiac MR, and all anti-angina medication was stopped 4 days before the examination. The use of Dipyridamole had to be stopped; yet, when this was not an option, it was considered a contraindication. From this prospective study, we retrospectively selected patients with no perfusion defects, and patients with only ischemic defects or only infarcts. A total of 64 patients were included: ten patients with ischemic myocardium, 15 patients with infarcted myocardium, and 39 control patients.

### Cardiac MR imaging protocol

All patients were scanned with a 1.5 T MR system (MAGNETOM Avanto; Siemens Healthineers, Erlangen, Germany). After the standard cine images were taken, a native Modified Look-Locker Inversion Recovery (MOLLI) based T1-mapping acquisition (WIP780B, Siemens Healthcare) was performed in short-axis views at both rest and stress. A 5(3)3 sampling scheme of the heart was performed, including eight images in 11 heartbeats. For the MOLLI acquisition, an initial inversion time of 110 ms was used with an 80 ms increment.

A single-shot steady-state free-precession readout T1 sequence (i.e. MOLLI) was used to acquire the images. Other parameters used in the image acquisition included the following: field of view, 300 × 256 mm^2^; slice thickness, 8 mm; acquisition matrix, 192 × 128; in plane spatial resolution, 1.4 × 1.4 mm^2^; bandwidth, 1085 Hz/pixel; flip angle, 35°; TR, 279 ms; TE, 1.1 ms; and parallel imaging acceleration factor, 2. Pixel-wise T1 maps of the myocardium were generated with inline motion correction. T1-maps were acquired at rest and at peak dose adenosine stress (140 µg/kg/min) in three short-axis slices (basal, mid-ventricular and apical). The mid-ventricular slice was used for analysis.

Stress-only perfusion imaging was performed as previously described in accordance with conventional methods [7]. A nonselective saturation recovery perfusion sequence was started during the first pass of contrast agent (0.1 mmol/kg gadopentetate dimeglumine) at peak dose adenosine infusion (140 µg/kg/min). The contrast was injected at a flow rate of 5 ml/s to ensure adequate flow. Equal position of the three short-axis slices was used in T1 mapping, stress perfusion, and late gadolinium enhancement (LGE). An additional dose of contrast material (0.05 mmol/kg) was given for LGE imaging. Conventional phase-sensitive inversion recovery LGE imaging was performed approximately 10–12 min after the stress series using the following parameters: field of view, 340 × 255 mm^2^; slice thickness, 8 mm; acquisition matrix, 192 × 144; in plane spatial resolution, 1.77 × 1.77 mm^2^; bandwidth, 1085 Hz/pixel; flip angle, 565°; TR, 2.6 ms; TE, 1.1 ms; and parallel imaging acceleration factor, 2.

### Image analysis

The T1-maps, generated on the imaging console, were analyzed on commercially available software (QMASS analytical software, Medis, Leiden, The Netherlands). Short-axis T1-maps were manually contoured using conservative septal sampling; specific sampling was used in ischemic or infarcted regions of interest (ROI) based on perfusion images and LGE images [8]. T1-reactivity was expressed as a percentage that was calculated according to the following:

$${\text{T}}1{\text{-reactivity}}\;(\% )=({\text{T}}1\,{\text{stress}} - {\text{T}}1\,{\text{rest)/T}}1\,{\text{rest}} \times 100$$

Patients were assigned to the control, ischemic, or infarcted group based on the results of their perfusion and LGE imaging. Patients were assigned to the control group if they presented no defects in both the perfusion series and the LGE series. Patients with a clear perfusion defect on the perfusion series, but without any defects on the LGE images, were assigned to the ischemic group.

To avoid partial volume effects of the blood pool, all samples were located in the core of the ROI. All infarcted and ischemic ROIs were placed away from the endocardial and epicardial borders. Remote myocardium was defined as myocardium without defects in the ischemic and infarcted group. Remote myocardial ROIs were placed carefully outside of the ischemic or infarcted region and if possible in the septal area were the normal myocardium was sampled.

### Statistical analysis

Continuous values were presented as mean value ± standard deviation (SD), or median with interquartile range (IQR). Categorical data were presented as numbers with a percentage. A Mann Whitney-U test was used to compare groups regarding data that was not normally distributed. Statistical analysis was performed using SPSS Statistics 23 (IBM Corporation, USA). A p-value < 0.05 was considered statistically significant.

## Results

The baseline characteristics and hemodynamics during the cardiac MR evaluation of all included subjects are presented in Table 1. The weight and BMI of the patients in the ischemic group was significantly higher (p value 0.003), resulting in the use of a significantly higher volume of contrast agent (p value 0.004). The infarcted group was composed of significantly less men than the control group (p value 0.004). Heart rate increased in all three groups with the use of adenosine; but heart rate increased significantly less during stress among participants in the infarcted group (p value 0.047) compared to those in the control group. The same trend was present in the ischemic group, but it was not of significant value.

**Table 1:** Patient characteristics

	Controls (N = 39)	Patients with ischemia (N = 10)	p value	Patient with infarction (N = 15)	p value
Male (n)	17 (44)	3 (30)	0.128	2 (13)*	0.004
Age (years)	66 (59–74)	73 (62–78)	0.274	73 (58–79)	0.069
BMI	25 (23–29)	29 (26–32)*	0.035	26 (24–30)	0.852
Hypertension	23 (59)	5 (50)	0.435	9 (60)	0.598
Hyperlipidemia	22 (57)	5 (50)	0.494	9 (60)	0.530
Diabetes	7 (18)	3 (30)	0.328	3 (20)	0.571
History of PCI [n (%)]	1 (3)	0	0.796	2 (13)	0.183
History of CABG [n (%)]	0	0	–	2 (13)	0.073
Resting HR (beats/min)	73 (66–79)	71 (63–79)	0.470	75 (63–95)	0.692
Stress HR (beats/min)	89 (75–100)	78 (69–91)	0.135	82 (70–101)	0.445
Increase in HR, beats/min (%)	15 (22)	11 (10)	0.517	10 (10)*	0.047
Rest SBP (mmHg)	143 (125–172)	150 (141–190)	0.320	152 (144–170)	0.258
Stress SBP (mmHg)	135 (116–153)	155 (130–177)	0.072	139 (130–149)	0.320
Change in SBP [mmHg (%)]	− 8 (6)	5 (3)	0.501	− 13 (9)	0.699
Rest DBP [mmHg]	81 (75–90)	81 (75–86)	0.634	81 (77–85)	0.862
Stress DBP [mmHg]	80 (72–86)	77 (73–87)	0.705	81 (75–87)	0.609
Change in DBP [mmHg (%)]	− 1(1)	− 4 (5)	0.398	0 (0)	0.677

Values are given as n(%) or as median (IQR)

*BMI* body mass index, *PCI* Percutaneous intervention, *CABG* coronary artery bypass graft, *HR* heart rate, *SBP* systolic blood pressure, *DBP* diastolic blood pressure

*p < 0.05 is significantly different compared to the control group

**Fig. 1:**
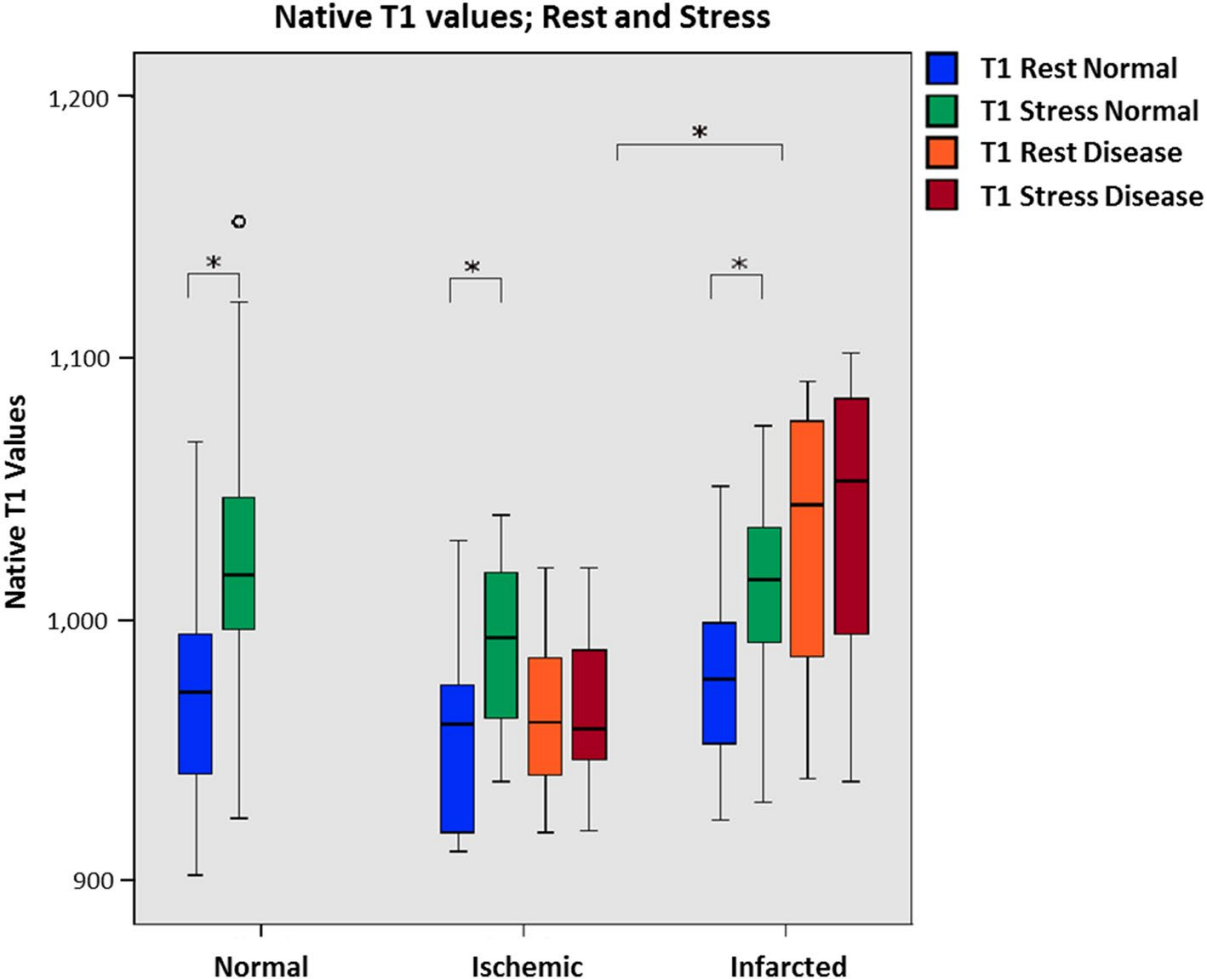
Boxplot of native T1-values at rest and stress (median; IQR) for the control group, the ischemic group and the infarcted group in normal/remote and diseased myocardium. The T1-values in infarcted myocardium are significantly higher than the T1-values in ischemic myocardium. During stress, the native T1-values increased compared to those during rest in normal and remote myocardium. While there was no significant increase in ischemic and infarcted myocardium. Asterisk indicates significant differences

In Fig. 1, the T1-values are presented for the three different groups. The native T1-values for normal myocardium were as follows: median, 972 ms; and IQR, 939–994 at rest. During stress, the normal myocardium T1-values were significantly higher (1017 ms; IQR 996–1049) than at rest (p value < 0.001). The rest T1-values for remote myocardium were 960 ms (IQR 918–982) and 977 ms (IQR 945–999) in the ischemic and infarcted group, respectively, showing a significant increase at stress (p value < 0.001 and 0.001). The native T1-values in infarcted myocardium were significantly higher compared to the value in ischemic myocardium during both rest and stress (p value 0.001 and 0.001); see Fig. 1. The resting native T1-values in infarcted myocardium were significantly higher than the native T1-values in normal myocardium among the control group (p value 0.003). The ischemic group showed no significant difference in native T1-values of the remote myocardium compared with the control group. Table 2 provides an overview of native T1 values for all groups.

**Table 2:** Native T1 and T1 reactivity values for normal, ischemic and infarcted myocardium

Status	Normal/remote myocardium			Diseased myocardium			Paired samples-test
	T1 rest	T1 stress	DeltaT1 normal/remote	T1 rest	T1 stress	DeltaT1 disease	
Control N = 39	972 (939–994)	1017 (996–1049)	4.15 (3.20–7.03)	–	–	–	
Ischemic N = 10	960 (918–982)	993 (961–1020)	3.54 (1.48–5.78)	961 (939–988)	958 (945–988)	0.00 (− 0.18 to 0.16)	0.000
Infarcted N = 15	977 (945–999)	1015 (991–1040)	3.21* (1.95–4.79)	1044* (985–1076)	1053*(989–1088)	0.41 (0.09 to 0.86)	0.001

Values are represented as medians with interquartile ranges (IQR)

*Indicates significant difference compared to the control group (p < 0.05) using the Wilcoxon paired sample test

An overview of T1-reactivity values for the different groups is given in Table 2. The T1-reactivity for remote myocardium in the infarcted group (3.21%; IQR 1.95–4.79) was significantly lower (p value 0.04) than the T1-reactivity in the control group (4.15%; IQR 3.20–7.03). There was a significant difference in T1-reactivity between ischemic and remote myocardium (0.00%; IQR − 0.18 to 0.16 and 3.54%; IQR 1.48–5.78, p value < 0.001), and between infarcted and remote myocardium (0.41%; IQR 0.09–0.86 and 3.21%; IQR 1.95–4.79, p value 0.001). No significant difference in T1-reactivity resulted between ischemic and infarcted myocardium.

**Fig. 2:**
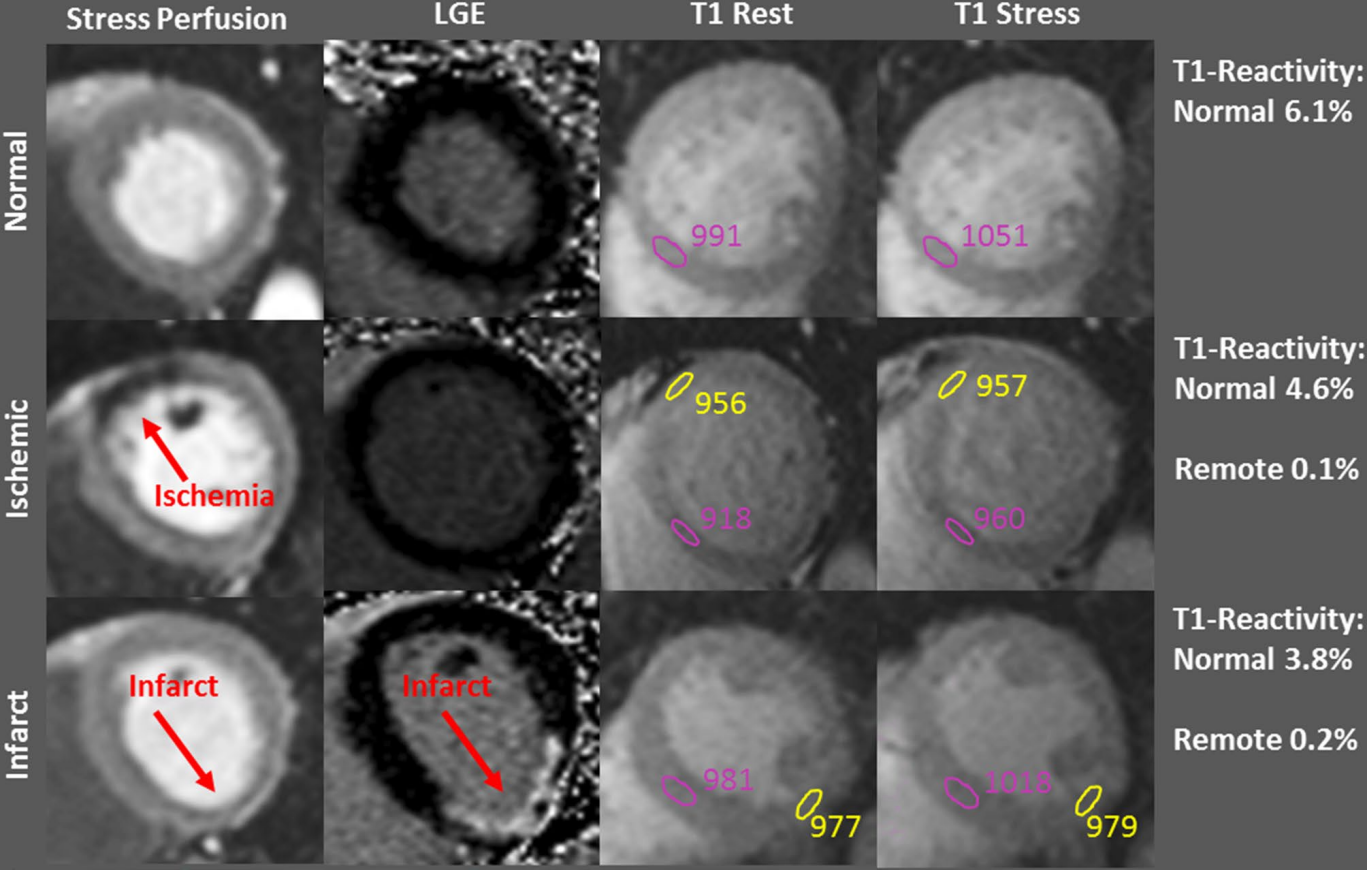
Examples of cardiac MR images for three patients including Adenosine Stress perfusion, LGE images and rest/stress T1 maps. The upper row shows a control patient without ischemic or infarcted myocardium. The middle row shows a patient from the ischemic group, with a perfusion defect (red arrow) on the stress perfusion images. The lower row shows a patient from the infarct group, with a defect on the LGE images (red arrow). Regions of interest in the T1 images are shown for diseased normal/remote myocardium (pink) and diseased myocardium (yellow)

Figure 2 shows examples of patients from the three groups with corresponding defects on the stress perfusion images, LGE images and rest/stress T1-mapping images.

## Discussion

This study demonstrates the possibilities for tissue characterization in cardiac MR using native T1-mapping at rest and stress among a population with intermediate-high risk for CAD. We show that T1-reactivity can discriminate between normal and diseased regional myocardium (ischemic and/or infarcted). However, there was no significant difference in T1-reactivity between ischemic and infarcted myocardium. Furthermore, native T1-values were significantly higher in infarcted myocardium at both rest and stress compared to ischemic myocardium; and could potentially be used to differentiate between ischemic and infarcted myocardium.

Native T1-values in normal and remote myocardium were within previously published ranges [4]. The T1-reactivity decreased in remote myocardium among the ischemic and infarcted group in comparison to the normal control group. This decrease in T1 reactivity could be an indication of coronary microvascular dysfunction, as suggested by Liu et al. and by Arnold et al. [9].

A previous study by Liu et al. reported on the ability of native T1-mapping to differentiate between normal, ischemic and infarcted myocardium [6]. An important difference between our study and the one completed by Liu et al. is the fact that they used an investigational shMOLLI T1 sequence compared to the MOLLI sequence that was used in our study. The decreased heart rate sensitivity of the shMOLLI sequence comes at the cost of significant loss of precision [10]. Due to the more established position of the MOLLI sequence in both research and clinical practice, we decided to use the MOLLI 5(3)3 scheme in our study. We were able to show that using this sequence also enabled the differentiation between normal, ischemic and infarcted myocardium. In contrast with the study by Liu et al., we did not find a difference in native T1-values between normal/remote and ischemic myocardium. A possible explanation for this could be the limited number of patients with ischemia included in this study. Increasing the sample size could result in significant results. A second reason could be thatin our study, we excluded ischemic patients with evidence of myocardial infarction. The combination of ischemic and infarcted myocardium in the same patient cohort could influence the results.

A study by Messroghli et al. showed similar native T1-values in infarcted myocardium, while Liu et al. showed much higher values [11]. Native T1-values are greatly influenced by differences in the applied T1-mapping sequence, scanner type and manufacturer. These components are different from the ones used in our study.

Since T1-values are representative for the water content of the tissue, it is expected that they are associated with MBV. The T1-reactivity reflects the underlying increase of MBV during stress, while the native T1-value is influenced by multiple other factors, such as edema and interstitial fibrosis. Studies on dynamic CT myocardial perfusion imaging have shown that MBV is not only able to discriminate between normal and diseased myocardium, but also between ischemic and infarcted myocardium. Myocardial blood flow (MBF) is decreased in both ischemic and infarcted myocardium; whereas, MBV is only decreased in infarcted myocardium [2].

In this study, compared to values at rest, we saw an increase in native T1-values during stress in normal and remote myocardium among all three groups. No increase was observed in ischemic and infarcted myocardium possibly because the capillary recruitment in these diseased areas is already maximized to compensate for the reduced MBF. As a direct consequence, the absence of T1-reactivity in diseased myocardium could be explained by the fact that inducing stress does not result in an increased capillary recruitment; as a result, the water content stays equal between rest and stress. Our results show that native T1-values in infarcted myocardium are higher than in normal/remote and ischemic myocardium. This represents the fibrotic tissue in infarcted myocardium, where the extracellular space is larger than in normal myocardium, allowing for an increased extravascular blood accumulation [12, 13]. These results indicate that native T1-values and T1-reactivity are able to make the same differentiation made by dynamic perfusion CT using MBV without the use of contrast agents used in perfusion imaging.

The use of gadolinium has been a big discussion point considering recent studies demonstrated that intravenous administration of gadolinium is associated with deposits in the brain [14, 15]. Acknowledging that the use of gadolinium could be unnecessary for Native T1-mapping, especially T1-reactivity, this technique is an interesting potential substitute for myocardial first pass perfusion and LGE in the detection of ischemic and infarcted myocardium. Native T1-values vary between field strength, scanner and mapping sequence [4]. However, T1-reactivity values are expected to be less susceptible to variation due to the relative nature of this parameter. Together with the discriminatory ability between normal and diseased myocardium makes it an interesting possible biomarker for tissue characterization in cardiac MR.

T1-values and T1-reactivity were evaluated in specific regions of interest that were identified based on visual analysis of the perfusion and LGE series. Liu et al. showed that segmental analysis of T1-reactivity is feasible, and that there were no significant differences between interslice and intersegment T1-reactivity values. In our study and in the study of Liu et al., ischemic and infarcted areas were also identified based on manually drawn ROI’s within perfusion and LGE scans [6]. Further studies should be performed to investigate whether detection of ischemic and infarcted myocardium is feasible on a segmental basis, excluding the need to manually draw ROI’s. Also, the diagnostic accuracy of native T1 and T1-reactivity for the detection of myocardial perfusion defects in a large cohort of patients should be tested.

This study has several limitations. We used data from patients that were prospectively enrolled in the PARAMETRIC study. However, the perfusion, LGE and native T1-mapping acquisitions were retrospectively analyzed for the purpose of this study. We introduced a selection bias by excluding patients with a combination of ischemia and infarction. However, this decision was based on the secondary outcome in order to be able to investigate the differences of native rest-stress T1-values and T1-reactivity in the remote myocardium of patients with normal, ischemic, and infarcted myocardium. The T1 mapping protocol we used was optimized to meet the acquisition requirements at all heart rate levels, however, this resulted in suboptimal image quality (192 vs. 256 acquisition matrix) in lower heart rate patients (< 90/min). Finally, our study population is relatively small and future research should focus on validating our results in a larger cohort of patients.

## Conclusion

In conclusion, native T1-mapping using a MOLLI 5(3)3 sequence during rest and stress can replicate the prior findings of ShMOLLI of the ability to distinguish between normal, remote, ischemic and infarcted myocardium using absolute and relative T1-value parameters and holds potential as an imaging biomarker for tissue characterization in MR without the use of gadolinium.
